# IKZF1 selectively enhances homologous recombination repair by interacting with CtIP and USP7 in multiple myeloma

**DOI:** 10.7150/ijbs.70960

**Published:** 2022-03-21

**Authors:** Meng Liu, Ying Zhang, Yunzhao Wu, Jin Jin, Yang Cao, Zhixiao Fang, Lou Geng, Li Yang, Miao Yu, Zhilei Bu, Yanjie Ji, Huizhuang Shan, Zhihui Zou, Ligen Liu, Yingying Wang, Youping Zhang, Yin Tong, Hanzhang Xu, Hu Lei, Wei Liu, Fenghou Gao, Yingli Wu

**Affiliations:** 1 Hongqiao International Institute of Medicine, Shanghai Tongren Hospital / Faculty of Basic Medicine, Key Laboratory of Cell Differentiation and Apoptosis of the Chinese Ministry of Education, Shanghai Jiao Tong University School of Medicine, Shanghai 200025, China.; 2 Shanghai General Hospital, Shanghai Jiao Tong University School of Medicine, Shanghai 200080, China.; 3 Department of Ultrasound, Second Affiliated Hospital of Zhejiang University, Hangzhou 310009, China.; 4 Department of Hematology, The Third Affiliated Hospital of Soochow University, Changzhou, Jiangsu Province 213003, China.; 5 Institute of Translational Medicine, Shanghai General Hospital, Shanghai Jiao Tong University School of Medicine, Shanghai 200080, China.; 6 Department of Oncology, Shanghai 9th People's Hospital, Shanghai Jiao Tong University School of Medicine, Shanghai 201999, China.; 7 Changhai Hospital, Naval Medical University (Second Military Medical University), Shanghai 200433, China.

**Keywords:** IKZF1, DNA repair, HR, USP7, multiple myeloma

## Abstract

**Rationale:** In multiple myeloma (MM), the activities of non-homologous end joining (NHEJ) and homologous recombination repair (HR) are increased compared with healthy controls. Whether and how IKZF1 as an enhancer of MM participates in the DNA repair pathway of tumor cells remains elusive.

**Methods:** We used an endonuclease AsiSI-based system and quantitative chromatin immunoprecipitation assay (qChIP) analysis to test whether IKZF1 is involved in DNA repair. Immunopurification and mass spectrometric (MS) analysis were performed in MM1.S cells to elucidate the molecular mechanism that IKZF1 promotes DNA damage repair. The combination effect of lenalidomide or USP7 inhibitor with PARP inhibitor on cell proliferation was evaluated using MM cells* in vitro* and* in vivo*.

**Results:** We demonstrate that IKZF1 specifically promotes homologous recombination DNA damage repair in MM cells, which is regulated by its interaction with CtIP and USP7. In this process, USP7 could regulate the stability of IKZF1 through its deubiquitinating activity. The N-terminal zinc finger domains of IKZF1 and the ubiquitin-like domain of USP7 are necessary for their interaction. Furthermore, targeted inhibition IKZF1 or USP7 could sensitize MM cells to PARP inhibitor treatment *in vitro* and *in vivo*.

**Conclusions:** Our findings identify USP7 as a deubiquitinating enzyme for IKZF1 and uncover a new function of IKZF1 in DNA damage repair. In translational perspective, the combination inhibition of IKZF1 or USP7 with PARP inhibitor deserves further evaluation in clinical trials for the treatment of MM.

## Introduction

The research on DNA damage repair and genome stability has entered a new era after a century of steady advancement. This field mainly explores the secrets of the structural integrity and functional coordination of genomic DNA. Its results not only promote clinical disease prevention and treatment options, but also strengthen precise treatment of cancer. In recent decades, several types of DNA damage such as point mutation, deletion, insertion, inversion or transposition 1, and double-strand break (DSB) have been elucidated, among which DSB is the biggest risk of causing genome instability 2. Two main types of DSB repair pathways have been reported, namely homologous recombination (HR) and non-homologous end joining (NHEJ) 3, 4. Compared with the NHEJ pathway, HR is more conservative and error-free, which is particularly important for tumor cells with highly unstable genomes.

Multiple myeloma (MM) is a highly heterogeneous, terminally differentiated plasma cell malignancy that accounts for 10% of all hematological malignancies and 1% of all cancers 5. MM is characterized by genomic instability and can produce monoclonal immunoglobulins 6. Despite recent advances in diagnosis and treatment, some patients still show short-term response to treatment, relapse, and short survival 7. Many efforts have been made to develop the evaluation of the therapeutic effect of myeloma patients based on NHEJ (WHSC1, XRCC5 (KU80), PNKP, POLL), HR (EXO1, BLM, RPA3, RAD51, MRE11A and ATM) genes in MM patients 8-12. In addition to ATM, POLL, and PNKP, these repair genes have increased expression in MM patients with poor prognosis 13. Targeting DNA DSB repair pathways is a very promising strategy to enhance the efficacy of current programs and reverse drug resistance, thereby improving the prognosis of myeloma patients. Therefore, if the key proteins and molecular mechanisms involved in the regulation of NHEJ and HR repair pathways can be clearly identified in MM, patients can benefit from targeted therapy or combination therapy with DNA damaging agents.

The* IKZF1* gene encodes a transcription factor that interacts with multiple proteins, such as recombination activating gene Rag1 and Rag2 14, that are essential for the development of hematopoietic differentiation and proliferation. *Ikzf1*-knockout mice show severe impairment of lymphoid development, as well as myeloid and erythroid differentiation 15. Dysregulation of IKZF1 is associated with a variety of cancers, including leukemia 16-19, multiple myeloma (MM) 20, 21 and some solid tumors 22, 23. Although IKZF1 is believed to be a tumor suppressor in many cases 24, it serves as a tumor enhancer in MM 20, 21. Hence, inducing the degradation of IKZF1 is considered a strategy to combat MM 25, 26. For example, immunomodulatory drugs (IMiDs), such as lenalidomide, can induce the degradation of IKZF1 by activating E3 ubiquitin ligase cereblon (CRBN), which in turn results in the downregulation of c-Myc and IRF4, and inhibits the proliferation of MM cells 20, 21, 27. In the neoadjuvant chemotherapy study of muscle-invasive bladder cancer with high tumor mutation burden, it was found that chromosome 7p12 amplification (including *IKZF1*) can predict that patients will not respond to treatment with a specificity of 100%, suggesting that IKZF1 may be involved in tumor cell DNA damage repair. Colorectal cancer brain metastasis whole exome sequencing and whole genome sequencing data analysis showed that colorectal cancer brain metastasis mutation related genes (including *IKZF1*) are related to homologous recombination defects (HRD) feature. Whether IKZF1 as an enhancer of MM participates in the DSB repair pathway of tumor cells needs to be explored urgently. This will help expand MM's DNA repair knowledge, and also open up a therapeutic strategy that precisely targets IKZF1 involved in DNA damage repair pathways in MM.

In the present study, for the first time, we reveal a novel role of IKZF1 in HR repair, which can be regulated by interacting with CtBP-interacting protein (CtIP) and ubiquitin-specific protease 7 (USP7). Moreover, we identify USP7 as the deubiquitinating enzyme for IKZF1, regulating the ubiquitination and stabilization of IKZF1. Furthermore, we show that in the presence of PAPR inhibitor, inducing the degradation of IKZF1 by inhibiting USP7 or activating CRBN results in a synthetic lethal effect in MM cells *in vitro* and *in vivo.*

## Methods

### Antibodies and Reagents

The sources of antibodies against the following proteins were as follows: IKZF1(14859, Cell Signaling Technology); USP7 (A700-072, Bethyl Laboratories); Lamin B (12586, Cell Signaling Technology); GFP (50430-2-AP, Proteintech); FLAG (30503ES20, Yeasen); UHRF1 (12387, Cell Signaling Technology); P5091 (882257-11-6, ChemShuttle); His (66005-1-Ig, Proteintech); Myc (2276, Cell Signaling Technology); Ub (10201-2-AP, Proteintech); VP1 (633419-42-0, Sigma); CPT (CSN16581, CSNpharm); Olaparib (CSN12345, CSNpharm); 4-OHT (CSN22105, CSNpharm); CtIP (GTX70264, GeneTex); RPA2 (A2189, abclonal); Phospho-RPA2-S4/S8 Rabbit pAb (AP1102, abclonal); RAD51 (ab133534, Abcam); lenalidomide (CSN11280, CSNpharm); HA (AE008, abclonal); Rabbit mAb (9532, Cell Signaling Technology); β -Actin-HRP (conjugated) (AB2001, Abways); Anti-rabbit IgG, HRP-linked Antibody (7074, Cell Signaling Technology); γH2AX (80312, Cell Signaling Technology); Anti-Alexa Fluor 555 (P0190, Beyotime Biotechnology).

### RNA interference

The following shRNAs from Sangong Biotech were used in this study: IKZF1 shRNA-1: 5′-GCATTTGGAAACGGGAATAAA-3′, IKZF1 shRNA-2: 5′-CTACGAGAAGGAGAACGAA AT-3′, IKZF1-3' UTR shRNA-1: 5′-GCCTATCAATCATTAAGGTCAT-3′, IKZF1-3' UTR shRNA-2: 5′-GCATTTGGAAACGGGAATAAA-3′, USP7 shRNA-1: 5′-CCTGGATTTGTGGTT ACGTTA-3′, USP7 shRNA-2: 5′-GTGTCCTATATCCAGTGTAAA-3′.

### Plasmids

IKZF1 cDNA and its mutants were subcloned into pMSCVpuro retroviral transfer vector (Clontech) to form the pMSCV-puro-Flag-IK1 plasmid, pMSCV-puro-Myc-IK1 plasmid and the pMSCV-puro-HA-IK1 plasmid. DUB plasmids were purchased from Addgene (Cambridge, MA, USA). His-Ubiquitin and HA-Ubiquitin plasmids were kindly provided by Professor Jian Huang (Shanghai Jiao Tong University School of Medicine, Shanghai, China). USP7 WT (USP7 wild-type) and USP7 C223S (USP7 catalytic mutant) were cloned into a pFLAG-CMV-4 vector. GFP-tagged USP7 constructs (WT or mutants) were kindly provided by Prof. Jing Liu (Central South University, Changsha, China). HA-ER-AsiSI plasmid was kindly provided by Prof. Lei Shi (Tianjin Medical University, Tianjin, China).

### Immunopurification and silver staining

Lysates from 5×10^7^ MM1.S cells were prepared by incubating the cells in 1 mL lysis buffer [20 mM Tris-HCl (pH 7.5), 150 mM NaCl, 0.1 mM EDTA, 0.2% Triton X-100] containing protease inhibitor cocktail (Roche). The supernatants were incubated with the indicated primary antibodies (anti-IKZF1 antibody or normal IgG) and Protein A/G Plus agarose beads (Santa Cruz) at 4 °C overnight. The resins were washed three times with buffer containing 20 mM Tris-HCl (pH 7.5), 150 mM NaCl, 0.1 mM EDTA, and 1.0% Triton X-100. The bound proteins were dissolved in 1×SDS loading buffer followed by silver staining with a fast silver staining kit (Beyotime). The distinct protein bands were retrieved and analyzed by LC-MS/MS.

### Immunofluorescence

Cells were fixed with 4% paraformaldehyde and permeabilized with 0.5% Triton X-100 in PBS. Samples were then blocked in 2% bovine serum albumin in PBS for 1 h at room temperature and stained with the appropriate primary and secondary antibodies coupled to Alexa Fluor 488 or 594 (Invitrogen, Thermo Fisher Scientific). The cell nuclei were counterstained with 4,6-diamidino-2-phenylindole (DAPI; Molecular Probes, Eugene, OR, USA). Confocal images were captured on a laser confocal microscope (Nikon, Nagoya, Japan).

### RNA extraction and quantitative real-time PCR

Total RNA was extracted with TRIzol reagent (Invitrogen), and cDNA was synthesized using a reverse transcriptase kit (Thermo Scientific, Waltham, MA, USA), followed by qRT-PCR analysis using SYBR-Green qPCR master mix (Thermo Scientific) and an ABI PRISM 7900 system (Thermo Scientific). The primer sequences were as follows: IKZF1 forward 5'-GCTGCCACAACTACTTGG AAAGC-3' and IKZF1 reverse 5'-AGTCTGTCCAGCACGAGAGATC-3'; β-actin forward 5'-CATCCTCACCCTGAAGTACCC-3' and β-actin reverse 5'- AGCCTG GATAGCAACGTACATG-3'.

### *In vitro* deubiquitination assay

For the *in vitro* deubiquitination assay, HA-IKZF1 was co-expressed with His-ubiquitin in HEK293T cells and purified using an anti-HA antibody and Protein A/G Plus agarose beads under denaturing conditions (50 mM Tris- HCl, pH 8.0; 50 mM NaCl; 10 mM DTT; 1 mM EDTA and 5% glycerol). Next, ubiquitinated-IKZF1 proteins were incubated with purified USP7 protein (SinoBiological Inc., Beijing, China) in deubiquitination buffer (1 mM EDTA-Na_2_, 0.5 mM DTT, 50 mM Tris-HCl, dH_2_O) at 37 °C for 2 h. The reaction was terminated by boiling the mixture in 5×SDS sample buffer for 7 min. Then, the samples were resolved on 10% SDS-PAGE gels, followed by western blotting analysis.

### Comet assay

Cells were resuspended in 0.5% LMP agarose (100 mg in 20 mL of PBS) to result in a concentration of 3×10^5^ cells/mL. 5-10 μL of cell suspension were added to the slide for comet assay. The slide was protected from light and incubated in cold, freshly made lysing solution (2.5 M NaCl, 100 mM EDTA, 10 mM Tris (pH 10.0)) at 4 °C for a minimum of 1 h. Then, the slide was incubated in alkaline buffer containing 300 mM NaOH and 1 mM EDTA for 20-60 min before electrophoresis. The electrophoresis was conducted at 25 V for 25 to 40 min in the electrophoresis buffer containing 300 mM NaOH and 1 mM EDTA. DNA damage was measured in terms of tail moments using the CometScore software (casplab_1.2.3b2).

### Chromatin immunoprecipitation assay (ChIP)

The ChIP assay was performed according to the manufacturer's instructions (P2078; Beyotime). Eluted DNA was purified with a PCR purification kit (D0033; Beyotime) and analyzed by qPCR. DSB1_FW: 5'-GATTGGCTATGGGTGTGGAC-3'; DSB1_REV: 5'-CATCCTTGCAAACCAGTCCT-3'; DSB2-FW: 5'-TTCCTGCAGCCTCATTTTCT-3'; DSB2-REV: 5'-TGATGATGCCTTTTCCCTTC-3'.

### HR assay

Cells stably expressing DR-GFP or EJ2-GFP were transfected with pCBA-I-SceI-RFP and HA-IKZF1. After 2 days, cells were harvested and analyzed by fluorescence-activated flow cytometry (FACS) to examine the proportion of GFP/ RFP-positive cells. Results were normalized to the control group.

### CD138^+^ primary MM cells and bonemarrow-MNCs

Patients and healthy volunteers were informed to sign the informed consent forms before sample collection. The study has been approved by the Ethics Committee of Shanghai Jiao-Tong University School of Medicine. Mononuclear cells (MNCs) were isolated from the bone marrow specimens using Stemcell Lymphoprep^TM^ (Stemcell). CD138^+^ cells of the active MM patients were obtained from the bone marrow samples using CD138^+^ microbeads (Stemcell).

### Synergy calculations

Synergy data were analyzed with online software (https://synergyfinder.fimm.fi/synergy). ZIP synergy scores over 10 were considered to be synergistic, ZIP synergy scores between 0 and 10 were considered to be additive, and ZIP synergy scores below 0 were considered antagonistic.

### Tumor xenograft

Experiments were performed under the approval of the Experimental Animal Ethical Committee at Shanghai Jiao Tong University School of Medicine. NCI-H929 or RPMI-8226 cells were injected subcutaneously into the flanks of 6-week-old male BALB/c-Nu or NOD/SCID (National Cancer Institute/National Institutes of Health) mice, respectively. A 100 μL mixture of 1×10^7^ cells with 50% growth factor reduced Matrigel (BD Biosciences) was injected to each mouse. Mice bearing tumors of about 100 mm^3^ were divided randomly into the control group or experimental group. Tumor volume was measured every two days using calipers and calculated using the formula of length × width^2^/2. Mice were sacrificed for tumor dissection 3 weeks after the start of treatment.

### IKZF1 protein purification

The overexpression plasmid 3×Flag-IKZF1-pLVX was transferred into HEK293s cells and cultured for 48 hours. IKZF1 were purified according to the instructions for protein purification (SIGMA-ALDRICH, Cat: F4799).

### GST pull-down assay

Purified GST-tagged proteins (1 µg, GST: Cat: 11213-HNAE, Sino Biological; GST-His-USP7: Cat: 11681-H20B1, Sino Biological) were immobilized on Glutathione Beads 4FF (SA010100) and equilibrated with PBS-T binding buffer (PBS, pH 7.4, 1% Tween 20). Immobilized proteins were incubated for 2 h at 4 ºC with 1 µg 3×Flag-IKZF1 protein. After washing with cold PBS (1% Triton), bound proteins were eluted and analyzed by Western blot.

### MEF cells

*Ikzf1* knockout and wild type embryonic mice at 14-16 days were taken and their viscera and limbs were removed. The tissue was digested with trypsin into single cells and cultured with DMEM (10 % FBS).

### Statistical analysis

All the experiments were repeated for 3 to 4 times, and the data were presented as the mean ± standard deviation (SD). Student's *t* test was used for the comparison between two groups. All data were analyzed utilizing GraphPad Prism 5.0., and *p* <0.05 was considered statistically significant (*p< 0.05; **p <0.01).

## Results

### IKZF1 is essential in regulating DNA end resection

**Figure 1:**
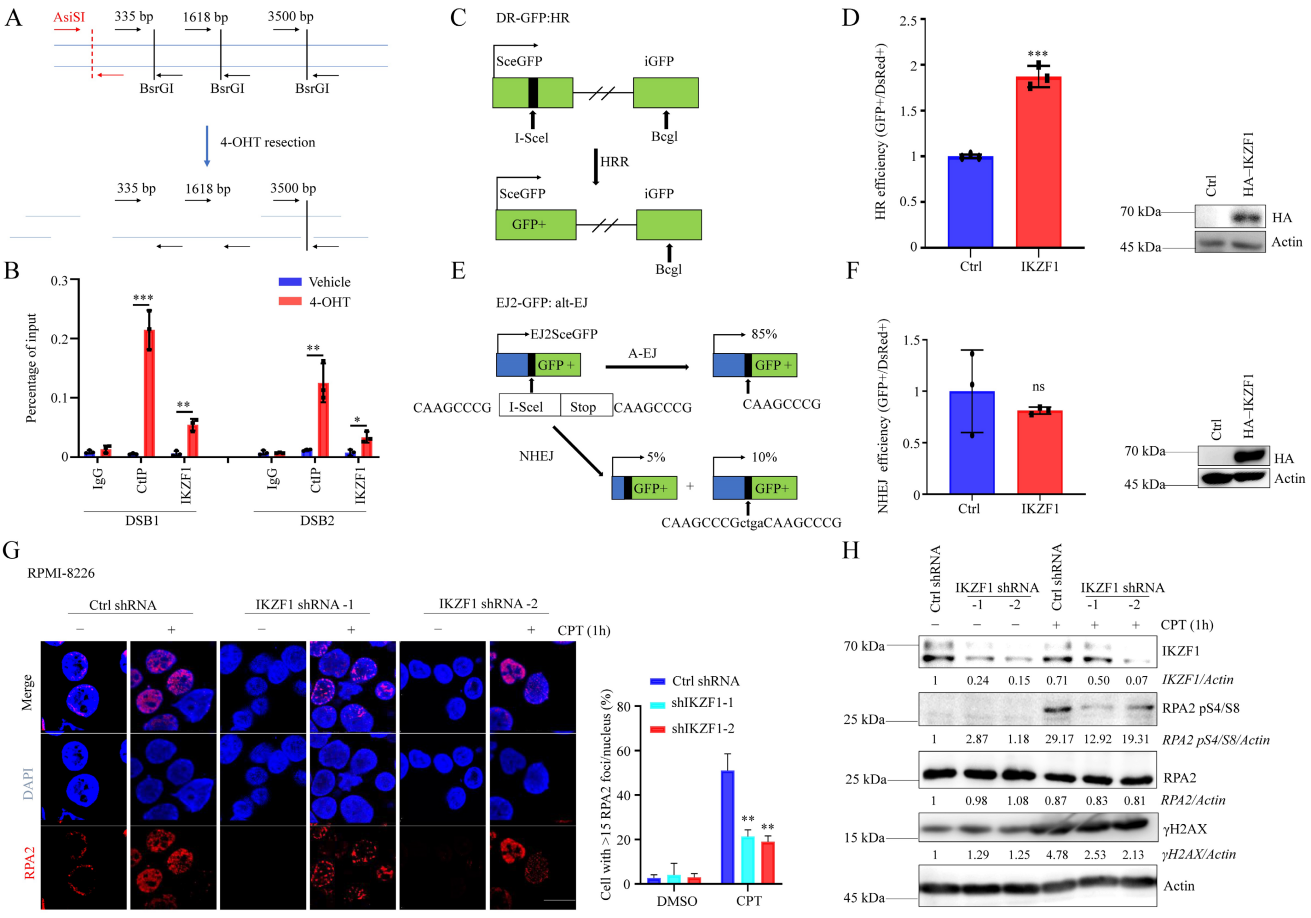
**IKZF1 regulates DNA end resection and HR. (A)** Illustration of the design of Taqman qPCR primers and probes (black arrows) for measuring resection at sites adjacent to the AsiSI sites (red arrows). The primer pairs are across BsrGI restriction sites. **(B)** qChIP analysis of IKZF1 recruitment around sites of DSBs. NCI-H929 cells stably expressing HA-ER-AsiSI were cultured in the absence or presence of 0.5 µM 4-OHT. qChIP experiments were performed using anti-IKZF1 or anti-CtIP antibodies with primers that covered the DNA sequences flanking the AsiSI cutting site and the distal region of the break. CtIP was used as a positive control. **(C-F)** Analysis of HRR **(C, D)** and alt-EJ **(E, F)** using the DR-GFP and EJ2-GFP reporter assay in U2OS cells showed that HRR is promoted by IKZF1 overexpression. **(G-H)** RPMI-8226 cells stably expressing control shRNA and IKZF1 shRNA were subjected to CPT (100 nM) for 1 h, the RPA2 foci of which were then examined. Scale bar: 20 µm **(G)**. The cellular extracts were prepared for western blotting with the indicated antibodies **(H)**. Data are mean ± s.d. p-values were analyzed by one-way analysis of variance (ANOVA) and two-sided Student's t-test. *p < 0.05, ns: no significant.

To clarify whether IKZF1 is participate in DNA repair, we decreased the IKZF1 in NCI-H929 cells, compared with the control group, cells transfected with IKZF1-specific shRNA became more sensitive to DNA damage agents such as olaparib, etoposide (VP16), and CPT (Fig. S1A, B and C). These data indicate that IKZF1 may be involved in DNA damage response. DNA double-strand break (DSB) is the most severe form of DNA damage. To investigate whether IKZF1 is recruited to DSB, we used an endonuclease AsiSI-based system (Fig. 1A), in which endogenous sequence-specific DSB could be generated in the presence of 4-hydroxyl-tamoxifen (4-OHT). Quantitative chromatin immunoprecipitation assay (qChIP) analysis revealed that IKZF1 was enriched around the DSB (CtIP was used as a positive control) (Fig. 1B). Next, we examined how IKZF1 promotes DNA repair using integrated reporter assays for HR (Fig. 1C) and NHEJ (Fig. 1E). We found that IKZF1 overexpression increased HR efficiency (Fig. 1D), but had no effect on NHEJ (Fig. 1F), suggesting that IKZF1 is involved in the HR pathway. In HR, short 3' single-stranded DNA (ssDNA) overhangs are firstly generated by the collaboration of by the CtIP endonuclease and the MRE11-RAD50-NBS1 (MRN) endo and 3' to 5' exonuclease complex. The 3′ ssDNA overhangs are bound by RPA, which is then displaced by RAD51 to form a RAD51-ssDNA nucleoprotein filament with the assistance of mediator proteins to facilitate HR. We find that IKZF1 depletion markedly decreased the formation of RPA2 foci (Fig. 1G), as well as the phosphorylation of RPA2 (RPA2 pS4/S8) (Fig. 1H), indicating that IKZF1 is essential in regulating DNA end resection.

### IKZF1 facilitates DNA repair

**Figure 2:**
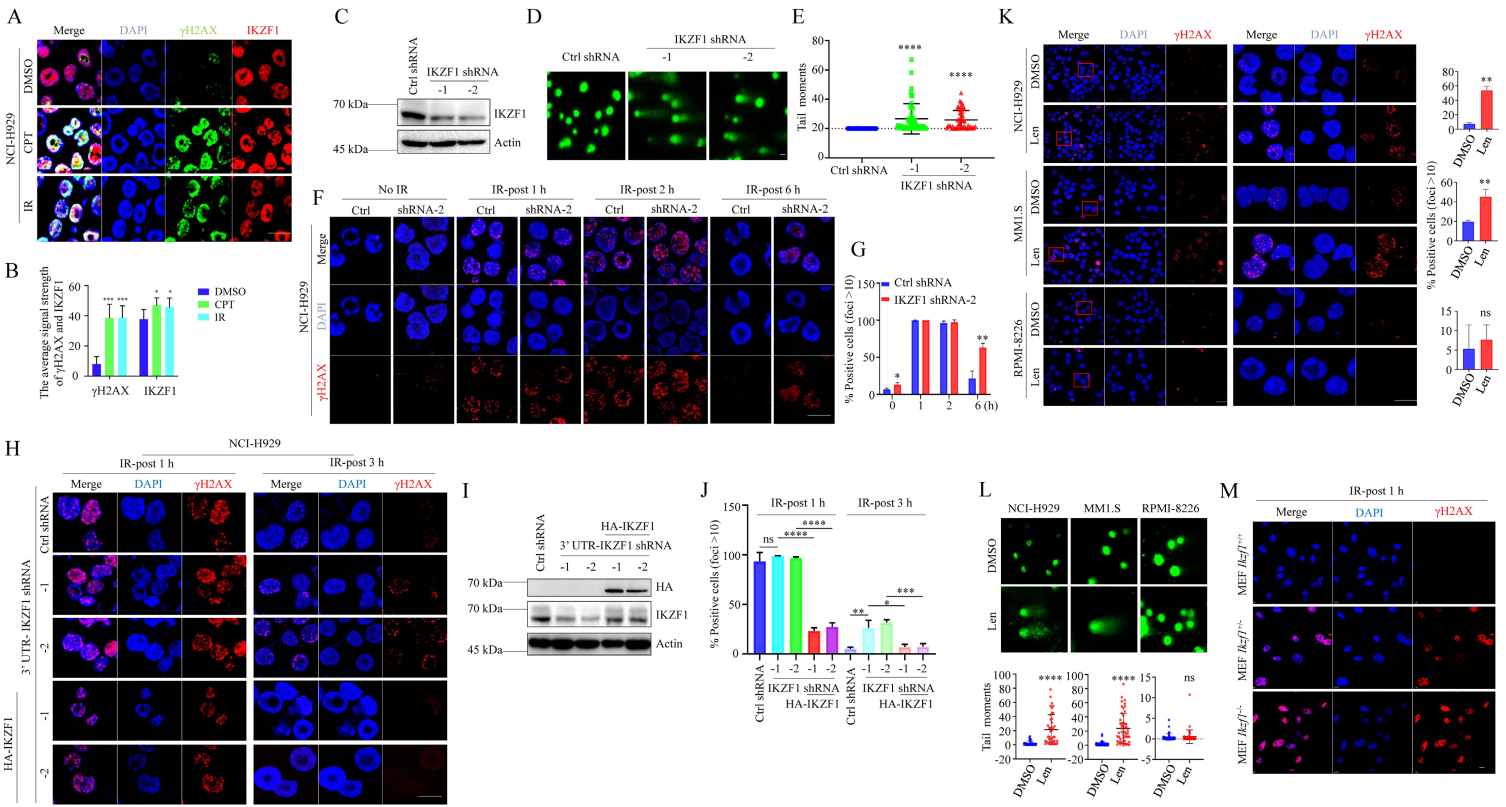
**IKZF1 is recruited to DSB sites and facilitates DNA repair. (A, B)** Confocal microscopic analysis of γH2AX foci formation upon DSBs. NCI-H929 were treated with CPT (100 nM) for 1 h or IR (5 Gy), fixed and immunostained with antibodies against IKZF1 and γH2AX. Scale bars: 20 µm. **(C-E)** NCI-H929 cells transfected with IKZF1 shRNAs **(C)**, DNA damage increased in IKZF1 shRNAs cells relative to control shRNA **(D)**, as quantified from comet assay-derived tail moments **(E)**. **(F, G)** NCI-H929 cells stably expressing control shRNA or IKZF1 shRNA were subjected to IR (5 Gy) and examined the γH2AX foci at the indicated time points. Scale bar: 20 µm. **(F)** Quantitation of γH2AX-positive cells (foci >10) is shown **(G)**. **(H-J)** IKZF1-KD NCI-H929 cells transfected with control vector, HA-IKZF1 (IKZF1/WT) **(I)** were subjected to IR (5Gy) and examined the γH2AX foci at the indicated time points. Scale bar: 20 µm **(H)**. Quantitation of γH2AX positive cells (foci >10) is shown **(J)**. **(K)** NCI-H929, MM1.S and RPMI-8226 cells were treated with 1 µM lenalidomide (Len) for 24 h, then fixed and immunostained with antibody against γH2AX (left panel). Scale bars: 20 µm. Quantitation of γH2AX positive cells (foci >10) is shown (right panel). The close-up views of areas indicated with red squares are shown in the right panels. **(L)** DNA damage was quantified from comet assay-derived tail moments. **(M)** MEF cells from *Ikzf^1+/+^*, *Ifzf^1+/-^* and *Ikzf^-/-^* mice were treated with IR (5 Gy). The cells were harvested after 1 h, fixed and immunostained with antibodies against IKZF1 and γH2AX. Scale bars: 20 µm. Data are mean ± s.d. *p*-values were analyzed by one-way analysis of variance (ANOVA) and two-sided Student's *t*-test. *p < 0.05, **p < 0.01, ***p< 0.001, ****p< 0.0001.

In NCI-H929 cells, two different DNA damage stimuli, ionizing radiation (IR) and camptothecin (CPT), resulted in the co-localization of IKZF1 and γH2AX (Fig. 2A, B). Similar results were obtained in U2OS cells overexpressed with IKZF1 (Fig. S2A). To provide direct evidence that IKZF1 responds to DNA damage at the single-cell level, we performed a comet assay in NCI-H929 cells transfected with IKZF1 shRNA or the control shRNA. Compared to the control group, knockdown of IKZF1 (Fig. 2C) caused a significant delay in DNA damage repair as measured by comet tail moment (Fig. 2D, E), suggesting that IKZF1 promotes DNA damage repair. Moreover, upon exposure to IR, knockdown of IKZF1 delayed the disappearance of γH2AX foci at 6 h (Fig. 2F, G, and Fig. S2B, C). To rule out the off-target effects of the IKZF1 shRNA, we ectopically expressed IKZF1 (WT) in IKZF1-knockdown cells. As shown in Fig. 2H-J, upon exposure to IR, knockdown IKZF1 delayed the disappearance of γH2AX foci at 3 h, and reconstitution with IKZF1 could reverse this effect. As expected, lenalidomide treatment can induce obvious DNA damage in NCI-H929 and MM1.S (lenalidomide-sensitive) cells with IKZF1 degradation but not in RPMI-8226 (lenalidomide-insensitive) cells with no IKZF1 degradation, as evaluated by the formation of γH2AX foci (Fig. 2K) and comet tail moments (Fig. 2L). The role of IKZF1 in DNA damage was further confirmed by using *Ikzf1*-knockout embryonic mouse fibroblast (MEF) cells (Fig. S2D and E). As shown in Fig. 2M and Fig. S2E, compared with the control cells, γH2AX foci increased in *Ikzf1*-knockout MEF cells upon IR treatment for 1 hour. Taken together, these results suggest that IKZF1 promotes DNA repair in MM.

### IKZF1 interacts with USP7

**Figure 3:**
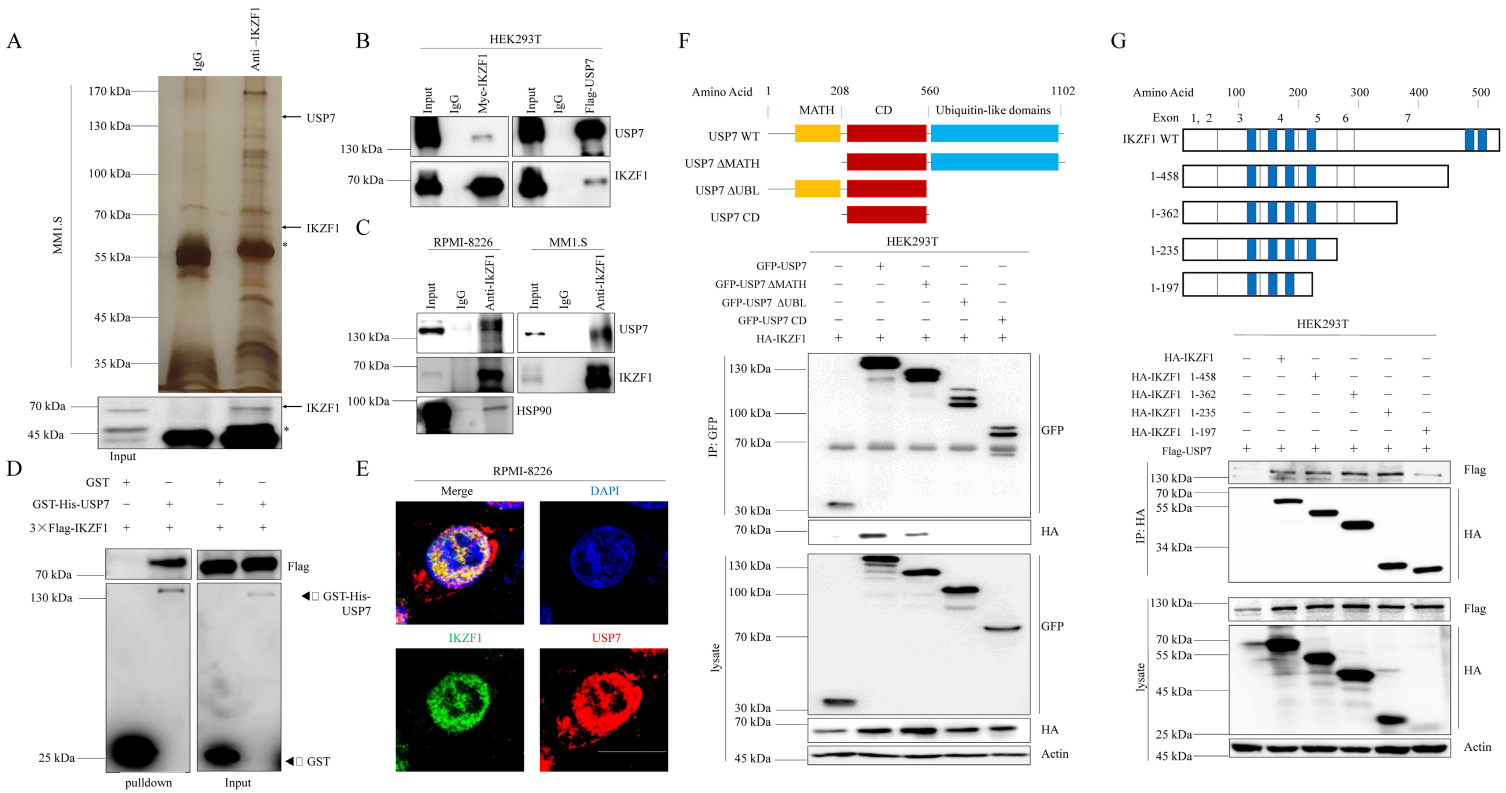
**IKZF1 is physically associated with USP7. (A)** Immunopurification and mass spectrometric analysis of IKZF1-interacting proteins. The asterisks denote the nonspecific IgG heavy-chain signal. **(B, C)** Co-IP analysis of the association between IKZF1 and USP7. Whole-cell lysates from HEK293T cells stably expressing Myc-IKZF1 an Flag-USP7, as well as RPMI-8226 and MM1.S cells were immunoprecipitated and immunoblotted with antibodies against the indicated proteins. **(D)** Detection of 3×Flag-IKZF1 bound to GST or GST-His-USP7 in a GST pull-down assay. **(E)** Confocal microscopic analysis of IKZF1 and USP7 subcellular localization. RPMI-8226 cells were fixed and immunostained with antibodies against the indicated proteins. Representative images from biological triplicate experiments are shown. Scale bars: 20 µm. **(F)** HEK293T cells stably expressing HA-IKZF1 were transfected with GFP-USP7 WT, GFP-USP7 ΔMATH, or GFP-USP7 ΔUBL, or GFP-USP7 CD, respectively. Cellular extracts were immunoprecipitated with anti-GFP antibody followed by immunoblotting (IB) with indicated antibodies. Domain names are: MATH: TRAF homology domain, CD: Catalytic domain, UBL: Ubiquitin-like domains. **(G)** HEK293T cells stably expressing Flag-USP7 were co-transfected with the indicated truncates of IKZF1, respectively. Cellular extracts were immunoprecipitated with anti-HA antibody followed by IB with indicated antibodies. Zinc fingers are depicted in blue boxes.

To elucidate the molecular mechanism that IKZF1 promotes DNA damage repair, we performed immunopurification and mass spectrometric (MS) analysis in MM1.S cells. Compared to normal IgG control, USP7 was identified as a potential interactor of IKZF1 (Fig. 3A). Co-IP assay was also performed to validate this result. Indeed, exogenous Myc-IKZF1 in HEK293T cells (Fig. 3B) and endogenous IKZF1 in MM cell lines (Fig. 3C) could pull down USP7, and reciprocal IP of exogenous USP7 effectively pulled down IKZF1 (Fig. 3B). GST pulldown assay shows that USP7 and IKZF1 can interact directly (Fig. 3D). Moreover, immunofluorescence assay showed that IKZF1 co-localizes with USP7 in the nucleus (Fig. 3E). We next mapped the binding region(s) between IKZF1 and USP7. As shown in Fig. 3F, the ubiquitin-like (UBL) domain of USP7 is required for its interaction with IKZF1. To map the critical domain of IKZF1 for its interaction with USP7, we used a series of IKZF1 truncating deletions (Fig. 3G) and several IKZF1 isoforms (IK2, IK6, and IK7) (Fig. S3). All bands for IKZF1 truncating deletions could be observed, while the IK2 isoform showed a weak band. Note that isoforms IK6 and IK7, in which the regions containing the first three zinc fingers are missing, showed no bands. The results showed that the N-terminal of IKZF1 (amino acid from 1 to 235), especially the region containing the second and third zinc fingers, are necessary for its interaction with USP7. These data suggest that USP7 directly interacts with IKZF1 between the ubiquitin-like domain of USP7 and the N-terminal region of IKZF1.

### USP7 stabilizes IKZF1 by regulating its ubiquitination

**Figure 4:**
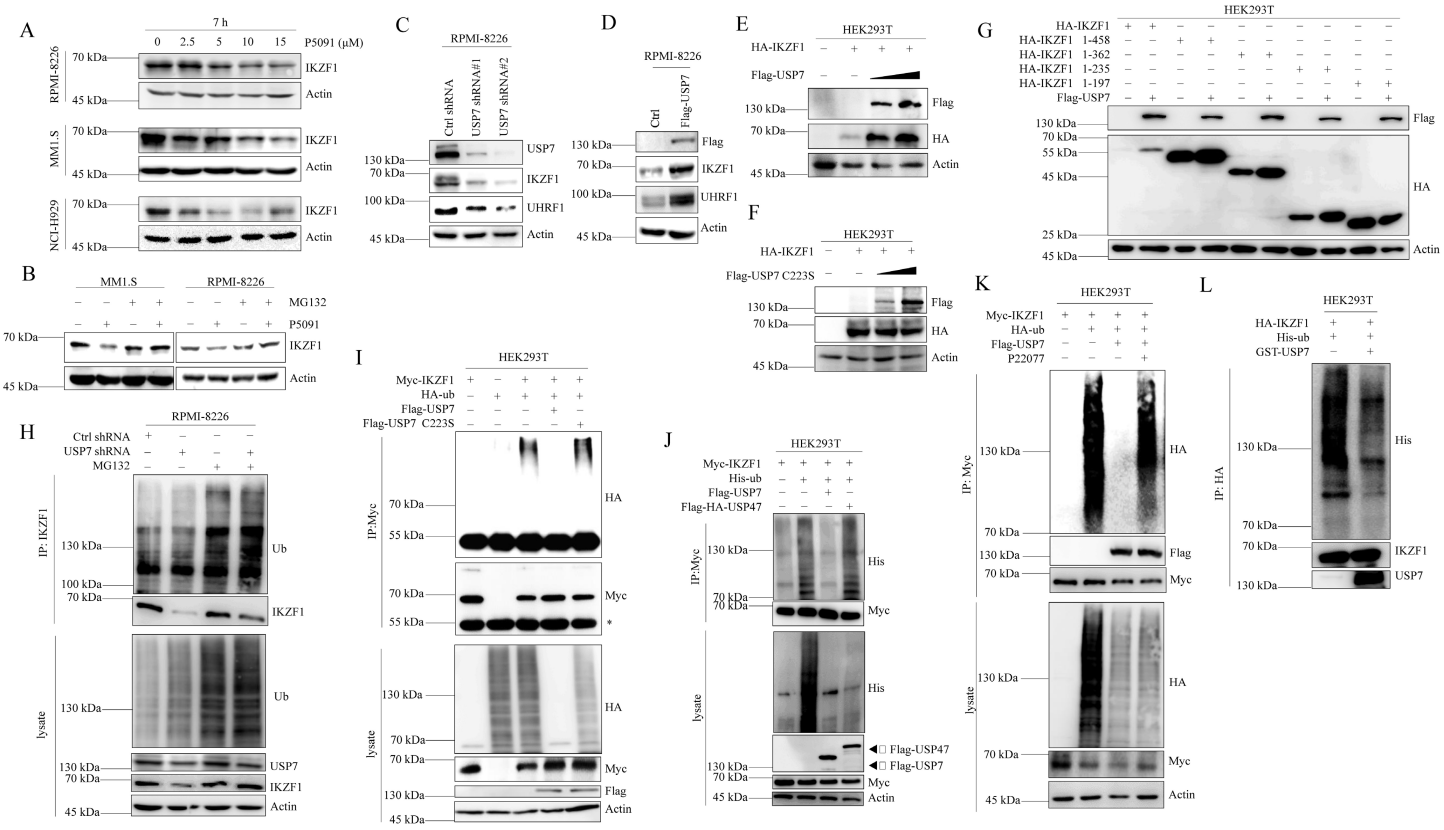
**USP7 deubiquitinates IKZF1. (A)** RPMI-8226, MM1.S, and NCI-H929 cells were cultured in the absence or presence of the indicated doses of P5091 for 7 hours. Cellular extracts were collected for western blotting with indicated antibodies. **(B)** RPMI-8226 and MM1.S cells were treated with P5091 (15 µM) for 6 h and further incubated with or without MG132 (5 µM) for another 3 h; the indicated proteins were examined by western blotting. **(C)** RPMI-8226 cells were transfected with the control shRNA or USP7 shRNAs, **(D)** or overexpressed with Flag-USP7. Cellular extracts were collected for western blotting with indicated antibodies. **(E-F)** HEK293T cells stably transfected with HA-IKZF1 were co-transfected with an increasing dose of Flag-USP7 **(E)** or its inactive C223S mutant **(F)**. Cellular extracts were collected for western blotting with the indicated antibodies. **(G)** HEK293T cells stably expressing Flag-USP7 were co-transfected with the indicated IKZF1 truncates, respectively. Cellular extracts were collected for western blotting with the indicated antibodies. **(H)** RPMI-8226 cells stably expressing control shRNA or USP7 shRNAs were treated with DMSO or MG132 (5 µM) for 3 h. Cellular extracts were immunoprecipitated with anti-IKZF1 followed by IB with indicated antibodies. **(I)** HEK293T cells stably expressing Myc-IKZF1 were co-transfected with HA-Ub/WT and Flag-USP7/WT or Flag-USP7/C223S as indicated. Cellular extracts were immunoprecipitated with anti-Myc antibody followed by IB with indicated antibodies. The asterisks denote the nonspecific IgG heavy-chain signal. **(J)** HEK293T cells stably expressing Myc-IKZF1 were co-transfected with His-Ub WT and Flag-USP7 WT or Flag-HA-USP47 as indicated. Cell lysates were subjected to western blotting with the indicated antibodies. **(K)** HEK293T cells stably expressing Myc-IKZF1 were co-transfected with HA-Ub/WT and Flag-USP7/WT and treated with P22077 (10 µM) for 6 h before being harvested. Then, cellular extracts were immunoprecipitated with anti-Myc antibody followed by western blotting with the indicated antibodies. **(L)** *In vitro* deubiquitination assays were performed with His-Ub-conjugated IKZF1 purified from HEK293T cells and recombinant GST-USP7/WT (0.5 µg) at 37 °C for 2 h, followed by western blotting with the indicated antibodies. Data are mean ± s.d. *p*-values were analyzed by one-way analysis of variance (ANOVA) **(A, C)** and two-sided Student's *t*-test **(D)**. ns: no significant.

We first examined whether USP7 inhibitor P5091 affects the stability of IKZF1 in MM cell lines. As shown in Fig. 4A, P5091 treatment reduced the IKZF1 protein level in a dose-dependent manner in different cell lines without affecting the mRNA level of IKZF1 (Fig. S4A). Moreover, P5091-induced reduction of IKZF1 can be rescued by proteasome inhibitor MG132 (Fig. 4B). These results indicate that inhibition of USP7 by P5091 induces the proteasome-mediated degradation of IKZF1. We then evaluated the effect of USP7 protein levels on IKZF1. As shown in Fig. 4C and Fig. 4D, knockdown or overexpression of USP7 in RPMI-8226 cell line results in IKZF1 protein decline or accumulation, respectively, without changing the mRNA level of IKZF1 (Fig. S4B and C). To further confirm this result, HEK293T cells were co-transfected with IKZF1 and different doses of USP7 (WT) plasmids. As expected, the overexpression of USP7 remarkably increased IKZF1 levels in a dose-dependent manner (Fig. 4E). Moreover, regulation of the IKZF1 protein level by USP7 is dependent on its DUB activity, as a catalytically inactive USP7 mutant (USP7 C223S) could not upregulate IKZF1 (Fig. 4F). Interestingly, the other three different DUBs inhibitors against USP2, or USP14, or USP9x did not decrease the levels of IKZF1 even at high doses that can cause cell death (Fig. S4D). To further test the selectivity of USP7 on IKZF1, we overexpressed a series of Flag-tagged DUBs in HEK293T cells stable transfected with Myc-IKZF1 and examined the protein level of IKZF1. The results showed that except USP7, none of the other tested USPs, such as USP47, USP2, USP5, and USP15, could increase the protein level of IKZF1 (Fig. S4E). We also investigated the interaction regions between USP7 and IKZF1 in cells. Consistent with the results from previous interaction experiments, USP7 can stabilize HA-IKZF1 1-458, HA-IKZF1 1-362, HA-IKZF1 1-235 (Fig. 4G) and Flag-IK2 (Fig. S4F). Also, GFP-USP7 (WT) and GFP-USP7 ΔMATH, but not GFP-USP7 ΔUBL or GFP-USP7-CD, could stabilize HA-IKZF1 (WT) (Fig. S4G). These results indicate that the stability of IKZF1 could be selectively regulated USP7. Next, we examined the effect of USP7 on the ubiquitination of IKZF1. As shown in Fig. 4H, compared to the control group, knockdown of USP7 in RPMI-8226 cells increased ubiquitin conjugation to IKZF1. Meanwhile, overexpression of wild-type USP7 but not USP7 C223S (Fig. 4I) or USP47 (Fig. 4J) removed the ubiquitination of IKZF1. Moreover, another USP7 inhibitor, P22077, also increased the ubiquitination of exogenous IKZF1 (Fig. 4K). To test whether USP7 directly removes polyubiquitin from IKZF1, we performed an *in vitro* deubiquitination assay by incubating purified ubiquitinated IKZF1 with recombinant USP7. Indeed, recombinant USP7 effectively removes ubiquitin from IKZF1, as demonstrated by the disappearance of ubiquitinated IKZF1 in Fig. 4L. These data suggest that USP7 can regulate the ubiquitination and stability of IKZF1.

### USP7 contributes to DNA repair through IKZF1

**Figure 5:**
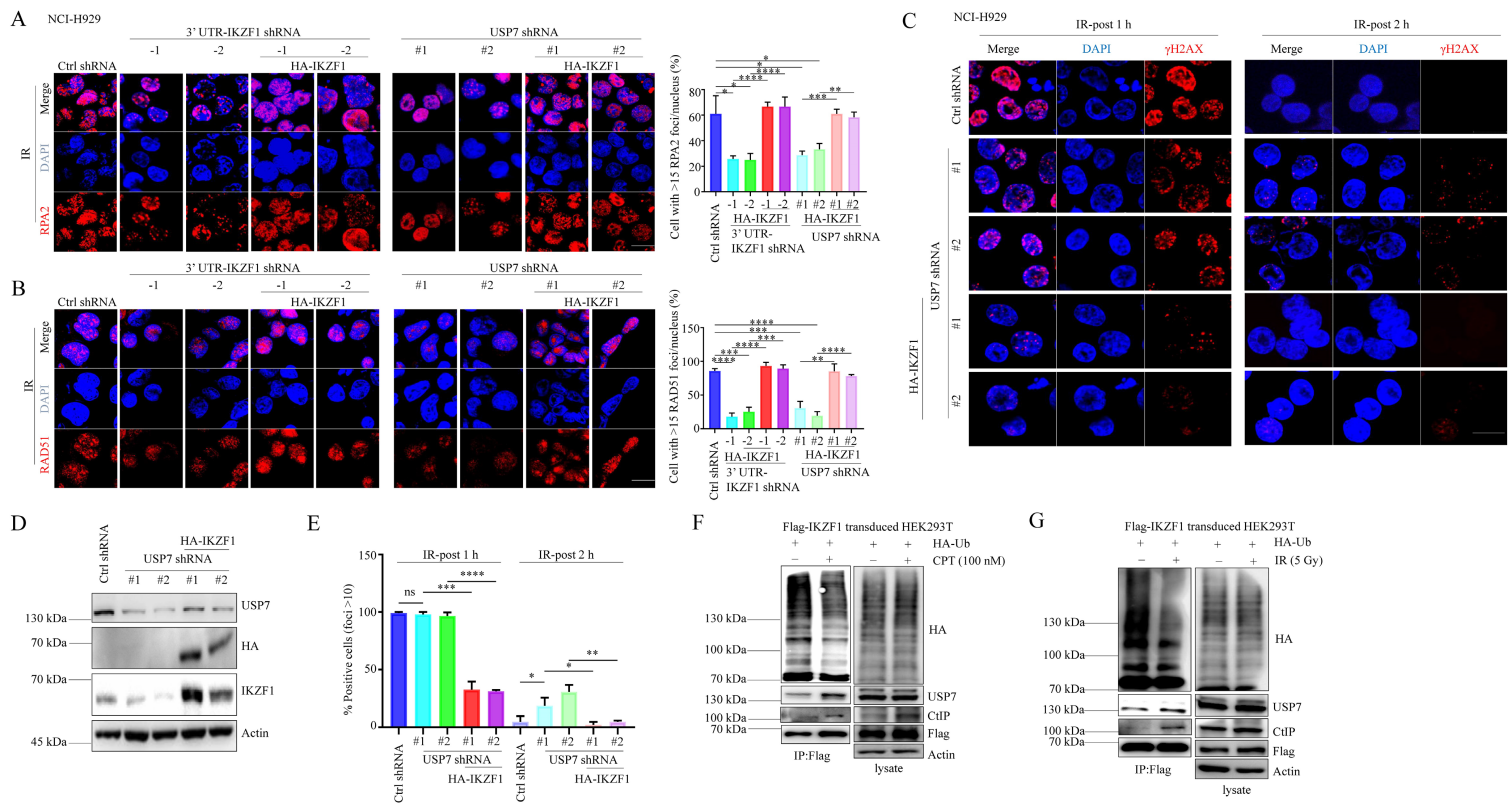
**USP7-mediated IKZF1 stabilization is potentiated by DNA damage. (A-B)** IKZF1-KD or USP7-KD NCI-H929 cells transfected with the control vector, HA-IKZF1 (IKZF1/WT) were subjected to IR (5 Gy) and examined for foci of RPA2 **(A)** and RAD51**(B)** by IF at the indicated time points. Scale bar: 20 µm. Quantitation of RPA2- or RAD51-positive cells (foci >15) is shown. Data are mean ± s.d. *p*-values were analyzed by one-way analysis of variance (ANOVA) and two-sided Student's *t*-test. *p < 0.05, ns: no significant. **(C-E)** USP7-KD NCI-H929 cells transfected with control vector, HA-IKZF1 (IKZF1/WT) were subjected to IR (5 Gy) and examined for the γH2AX foci at the indicated time points. Scale bar: 20 µm. Quantitation of γH2AX positive cells (foci >10) is shown. **(F-G)** HEK293T cells stably expressing Flag-IKZF1 were co-transfected with HA-Ub/WT followed by CPT (100 nM) for 1 h **(F)** or IR (5 Gy) **(G)** treatment. Cellular extracts were immunoprecipitated with anti-Flag antibody and then immunoblotted with the indicated antibodies. Data are mean ± s.d. *p*-values were analyzed by one-way analysis of variance (ANOVA). *p < 0.05, **p < 0.01, ***p< 0.001, ****p< 0.0001, ns: no significant.

Since several reports have demonstrated the role of USP7 in HR, we asked whether IKZF1 is involved in USP7-mediated HR. To this end, we overexpressed IKZF1 in USP7-knockdown cells. In response to IR treatment, knockdown of USP7 results in the decrease of RPA2 and RAD51 foci formation (Fig. 5A and B), which could be alleviated by the overexpression of IKZF1. Moreover, in response to IR, knockdown USP7 delayed the disappearance of γH2AX foci at 2 h, which could be reversed by the overexpression of IKZF1 (Fig. 5C-E). These results suggest that IKZF1 potentially involves in USP7-mediated HR in cells. Based on these observations, we further investigated the dynamic interaction between USP7 and IKZF1 upon genotoxic insults. For this purpose, MM1.S and NCI-H929 cells were either exposed to IR or CPT, and examined at different time points. Interestingly, the protein (Fig. S5A and 5C) but not mRNA (Fig. S5B and 5D) levels of IKZF1 increased at earlier time points after IR or CPT treatment, which could be abrogated upon USP7 knockdown (Fig. S5E and F). These results indicate that USP7 regulates the abundance of IKZF1 in response to DNA damage. Moreover, we found that the interaction of IKZF1 with USP7 or CtIP was enhanced upon CPT (Fig. 5F) or IR treatment (Fig. 5G). Meanwhile, USP7 could remove polyubiquitin chains from IKZF1 upon DNA damage (Fig. 5F and G). These results indicate that USP7 possibly regulates the activity of IKZF1 in HR by deubiquitination.

### Targeted inhibition of USP7 and IKZF1 increases the sensitivity of MM cells to PARP inhibitor* in vitro* and *in vivo*

**Figure 6:**
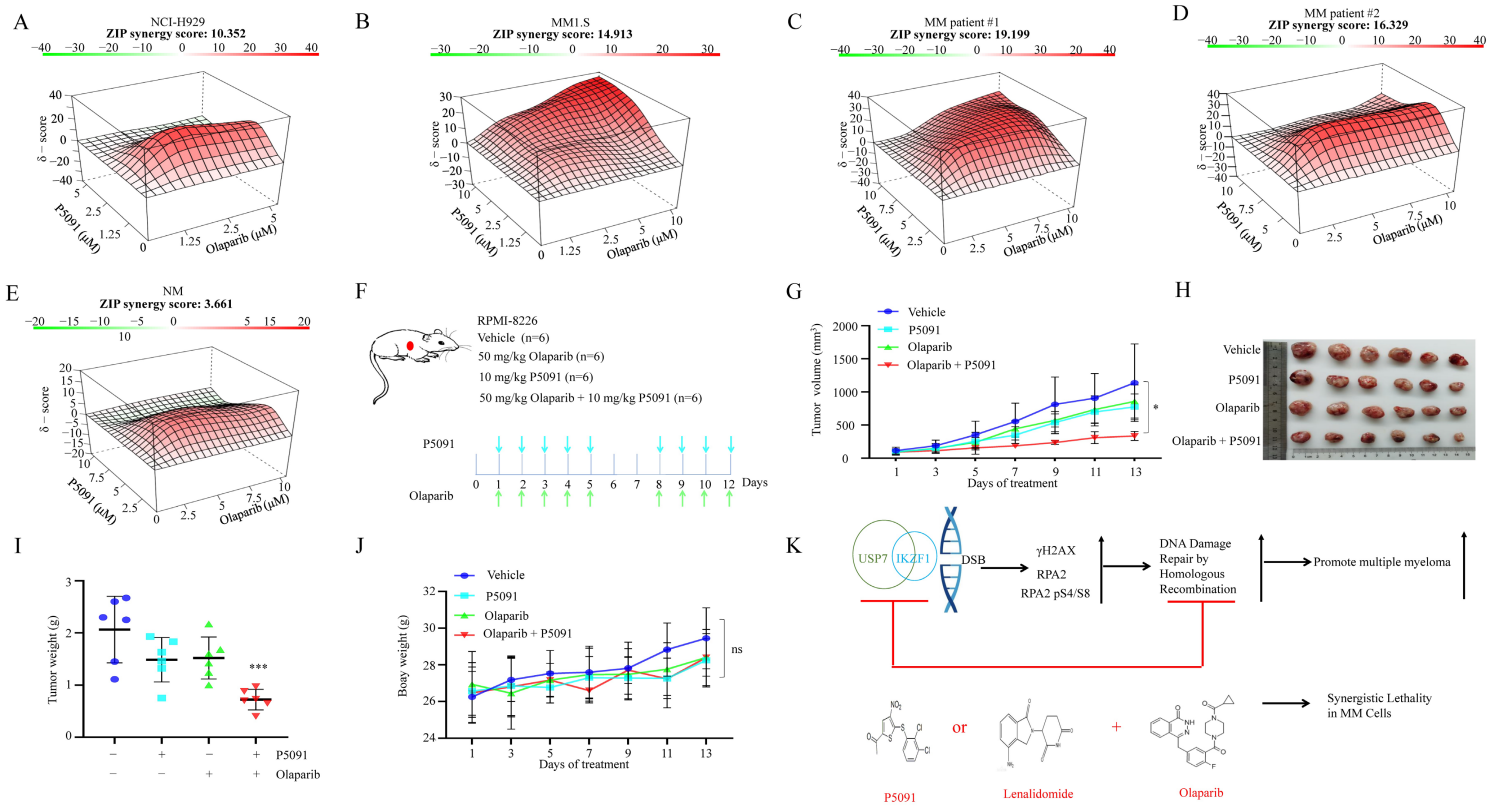
**P5091 sensitizes multiple myeloma cells to Lenalidomide *in vitro* and *in vivo*. (A-E)** NCI-H929 **(A)**, MM1.S **(B)**, CD138+ MM patient cells **(C, D)**, and normal BM mononuclear cells **(E)** were cultured in the control medium or in the presence of P5091 and/or olaparib for 48 h. The cell viability was determined by the CCK-8 kit. Data were analyzed online (https://synergyfinder.fimm.fi). **(F-J)** RPMI-8226 cells were subcutaneously injected into the flank of NOD-SCID mice. Mice were treated with the vehicle, P5091 (10 mg/kg i.v.) and/or olaparib (50 mg/kg i.p.) **(F)**. Mice tumor volume **(G)**, tumor images **(H)**, tumor weight **(I)**, and body weight **(J)** were then assessed. **(K)** Lenalidomide or USP7i sensitizes multiple myeloma cells to PARPi *in vitro* and *in vivo*. USP7i: USP7 inhibitor; PARPi: PARP inhibitor. Data are mean ± s.d. *p*-values were analyzed by two-way analysis of variance (ANOVA). *p < 0.05, ***p < 0.001, ns: no significant.

Provided that knockdown of *IKZF1* impairs HR, and disruption of which can sensitize cells to DNA damage insults, we investigated whether reducing IKZF1 by lenalidomide or USP7 inhibitor could synergize with PARP inhibitor (PARPi) to suppress the proliferation of MM cells. For this purpose, we treated NCI-H929 and MM1.S cells with lenalidomide in the presence or absence of olaparib. As the results showed, co-treatment of lenalidomide with olaparib synergistically inhibited the proliferation of the NCI-H929 (Fig. S6A) and MM1.S (Fig. S6B) cells but not normal BM mononuclear cells (Fig. S6C). The combination of lenalidomide or P5091 with olaparib can induce apoptosis (Fig. S6D). More importantly, by using the MM xenograft model, we demonstrated that the combination of lenalidomide with olaparib (Fig. S6E) could significantly reduce tumor size (Fig. S6F-H), inhibit cell proliferation (S6J, K, Ki67 staining) compared with using either drug alone, but has no significant effect on the body weight (Fig. S6I). A similar synergistic effect was observed for the combination of P5091 and olaparib. As shown in Fig. 6, co-treatment of P5091 with olaparib synergistically inhibited the proliferation of NCI-H929 (Fig. 6A), MM1.S (Fig. 6B), and primary CD138^+^ MM cells (Fig. 6C-D), but not normal BM mononuclear cells (Fig. 6E). The combination of P5091 and olaparib (Fig. 6F) also exerted a synergistic effect in lenalidomide-insensitive RPMI-8226 cells* in vivo* (Fig. 6G-I) without changing body weight significantly (Fig. 6J). All of the data indicate that reducing IKZF1 increases the sensitivity of MM cells to PARPi *in vitro* and* in vivo* (Fig. 6K).

## Discussion

One important finding is that IKZF1 plays a role in DNA damage repair. As a transcription factor, the well-known function of IKZF1 is to activate or repress the expression of genes in lymphoid cell development and maturation 23. Our data reveal a novel function of IKZF1, namely regulating DNA damage repair. Several lines of evidence supported this notion: knockdown or inducing degradation of IKZF1 results in the increase of γH2AX foci formation in myeloma cell lines; deletion of IKZF1 also causes the increase of γH2AX foci formation in MEF cells; integrated reporter assays showed that IKZF1 is involved in HR. This finding means that IKZF1 may have a dual role in clinical settings. On the one hand, it is known that the impairment of HR may result in genome instability and enhance the development of cancer 28. In BCR-ABL-positive acute lymphoblast leukemia cells, the association of deletion of IKZF1 with adverse outcome may be due to the impairment of HR. On the other hand, downregulation of IKZF1 can also increase the sensitivity of cells to DNA damage drugs, exerting a synthetic lethality effect. It is possible that BCR-ABL-positive acute lymphoblast leukemia cells with IKZF1 deletion are more vulnerable to DNA damage agents such as PARPi. Therefore, the reduction of IKZF1 protein may function as a double-edged sword in the pathogenesis of cancers. In myeloma, the use of lenalidomide may impair DNA repair and also increase the sensitivity of cells to DNA damage insults.

The choice of DSB repair route depends on whether end resection occurs. If the end resection process is blocked, the only repair route available is NHEJ. If end resection occurs, the HR and NHEJ repair pathways compete to repair the lesion. A key participant in the HR mechanism produces a 3′single-stranded DNA tail stabilized by replication protein A (RPA), and CtIP promotes HR by initiating DSB end resection and ssDNA formation, leading to the DDR repair pathway tending to HR. The overactivation of 53BP1 leads to the instability of the genome of BRCA1^-/-^ mice by inhibiting the HR pathway selection. In BRCA1 knockout cells, 53BP1 can end the excision by blocking CtIP at the DNA end, leading to NHEJ pathway selection. Consistent with a previous report 29, we showed that IKZF1 interacts with CtIP in MM cells and promotes DSB end resection. This may be the main reason why IKZF1 participates in the DDR pathway of HR without involving the NHEJ pathway in MM cells. In fact, it has been found that in the MM cell line, the increased HR efficacy causes the cells to develop resistance to dexamethasone and increase genomic instability. We found that IKZF1 specifically regulates the repair of HR pathway in MM cells, and supports a therapeutic strategy that targets IKZF1 to inhibit HR DNA damage repair pathway in MM. In support of this idea, IKZF1 mutations elevates mutational signatures of homologous recombination deficiency (HRD) and mismatch repair deficiency (MMRD) in colorectal cancer 30. In addition, Because IKZF1 is a transcription factor, we don't rule out the possibility that IKZF1 may regulate DNA repair at the transcriptional level. Indeed, in adult B-cell acute lymphoblastic leukemia (ALL), patients carrying IKZF1 deletion vs those without showed a unique signature featured by down-regulation of B-cell lineage and DNA repair genes 31.

IKZF1 contains seven exons that can give rise to multiple isoforms by alternative splicing. The N-terminal region of IKZF1 has a critical DNA-binding domain consisting of four zinc fingers, and the C-terminal region has a homo- and heterodimerization domain consisting of two zinc fingers. We and others have demonstrated that the E3 ubiquitin ligase CRBN and CHIP could lead to the ubiquitination of IKZF1 20, 21. However, the deubiquitinating enzyme against IKZF1 is not known. In this study, we identified USP7 as a bona fide DUB for IKZF1, which is supported by the following evidence: USP7 interacts and co-localizes with IKZF1 in MM cells; USP7 can remove the ubiquitin from IKZF1; inhibition of USP7 induces degradation of IKZF1 while overexpression of USP7 stabilizes IKZF1. Moreover, inhibitors for USP2, USP8, USP9X and USP1 or overexpression of USP2, USP5, USP15, USP47, etc (Supplementary Fig. S1A) could not change the stability of IKZF1, indicating the interaction between USP7 and IKZF1 is relatively specific. This finding reveals a new approach to induce the degradation of IKZF1, especially in those patients with lower expression or mutation of CRBN.

USP7, also known as herpesvirus-associated ubiquitin-specific protease (HAUSP), belongs to the USP subfamily of DUBs 32. USP7 has attracted much attention because of its role in various cancers 33. Interestingly, accumulating evidence shows that USP7 is a key player in DNA damage repair 34-38. By regulating the stability of DNA damage-associated proteins such as NBS1 35, MDC1 35, RNF168 38, RAD18 37, and CHK1 37, USP7 can regulate both the homologous recombination (HR) and non-homologous end joining (NHEJ) DNA-repair processes 39. USP7 is involved in the cell proliferation and cell death of MM 40, 41, by stabilizing c-Maf 41, NEK2 40, and HDM2 42. However, whether and how USP7 regulates DNA damage response in MM is not clear. Our data showed that IKZF1 is a novel player in USP7-mediated DNA repair. Moreover, in response to DNA insults, the interaction of IKZF1 with CtIP and USP7 increased. Considering the finding that MRN interacts with USP7 and CtIP, and overexpression of IKZF1 could partially abrogate the DNA damage induced by USP7 knockdown, we proposed that in response to DNA damage, USP7 may stabilize MDC1 and IKZF1, which further amplifies and sustains damage-sensing and repair signals. However, whether IKZF1 is also associated with the MRN-MDC1 complex still needs further investigation.

Inducing “synthetic lethality” represents a novel therapeutic strategy that selectively induce cell death of tumor cells 43. PARPi are the first clinically approved drugs designed to exploit synthetic lethality 44. PARPi are used routinely in the treatment of patients with HR-based DNA repair pathway deficits. For example, tumors arising in patients who carry germline mutations in either BRCA1 or BRCA2 are sensitive to PARPi because they have a specific type of DNA repair defect 45. In MM, a previous report showed that proteasome inhibitor could induce “BRCAness” state and results in a contextual synthetic lethality when combined with PARPi 46. In this paper, we show that the depletion of IKZF1 can impair HR and sensitize MM cells to PARPi *in vitro* and *in vivo*. Since lenalidomide and olapanib have been used clinically, our data support the combination of these two drugs in clinical trials in MM patients. Moreover, because IKZF1 cannot be effectively degraded in lenalidomide-insensitive RPMI-8226 cells, while it can be degraded by USP7 inhibitor, the combination of USP7 inhibitor with PARPi may provide an alternative way to overcome lenalidomide resistance.

In conclusion, we revealed a novel role of IKZF1 in DNA repair. That is, upon DNA insults, IKZF1 is stabilized by USP7 and recruits CtIP to the DNA damage sites, where to perform end resection and HR repair. Moreover, we demonstrate that IKZF1 is a novel substrate of USP7 and inactivation of USP7 results in the degradation of IKZF1. We further demonstrated that the combination of the PARP inhibitor with IKZF1 degraders such as USP7 inhibitor or lenalidomide, exerts a synthetic lethal effect on MM cells *in vitro* and *in vivo*. Our data suggest that IKZF1 depletion may serve as a biomarker of PARPi sensitivity and the combination of IKZF1 degraders with PARPi warrants further investigation in future clinical trials.

## Supplementary Material

Supplementary figures.
