# Supplementary material for: IKZF1 selectively enhances homologous recombination repair by interacting with CtIP and USP7 in multiple myeloma
Source: Int J Biol Sci. 2022 Mar 21;18(6):2515–26. doi: 10.7150/ijbs.70960 (PMC8990476; doi:10.7150/ijbs.70960)
Supplement: Supplementary file 1 — Supplementary figures. [file ijbsv18p2515s1.pdf]

Supplementary Materials for

IKZF1 selectively enhances homologous recombination repair by interacting with CtIP and USP7 in multiple myeloma

Meng Liu[1#], Ying Zhang[2#], Yunzhao Wu[1], Jin Jin[3], Yang Cao[4], Zhixiao Fang[5], Lou Geng[6], Li Yang[1], Miao Yu[1], Zhilei Bu[7], Yanjie Ji[1], Huizhuang Shan[1], Zhihui Zou[1], Ligen Liu[1], Yingying Wang[1], Youping Zhang[1], Yin Tong[2], Hanzhang Xu[1], Hu Lei[1], Wei Liu[2*], Fenghou Gao[6*], Yingli Wu[1*]

[1] Hongqiao International Institute of Medicine, Shanghai Tongren Hospital / Faculty of Basic Medicine, Key Laboratory of Cell Differentiation and Apoptosis of the Chinese Ministry of Education, Shanghai Jiao Tong University School of Medicine, Shanghai 200025, China

[2] Shanghai General Hospital, Shanghai Jiao Tong University School of Medicine, Shanghai 200080, China

[3] Department of Ultrasound, Second Affiliated Hospital of Zhejiang University, Hangzhou 310009, China

[4] Department of Hematology, The Third Affiliated Hospital of Soochow University, Changzhou, Jiangsu Province 213003, China

[5] Institute of Translational Medicine, Shanghai General Hospital, Shanghai Jiao Tong University School of Medicine, Shanghai 200080, China.

[6] Department of Oncology, Shanghai 9th People's Hospital, Shanghai Jiao Tong University School of Medicine, Shanghai 201999, China.

[7] Changhai Hospital, Naval Medical University (Second Military Medical University), Shanghai 200433, China

[#] These authors contributed equally to this work

***Corresponding author**

Correspondence to:

Yingli Wu, M.D., Ph.D., Hongqiao International Institute of Medicine, Shanghai Tongren Hospital/Faculty of Basic Medicine, Chemical Biology Division of Shanghai Universities E-Institutes, Key Laboratory of Cell Differentiation and Apoptosis of National Ministry of Education, Shanghai Jiao Tong University School of Medicine, Shanghai 200025, China. Phone: 0086-21-63846590-776916; Fax: 0086-21-64154900, E-mail: wuyingli@shsmu.edu.cn;

Fenghou Gao, Ph.D., Department of Oncology, Shanghai 9th People's Hospital, Shanghai Jiao Tong University School of Medicine, Shanghai, 201999, China. Phone: 0086-21-56691101-6964; Fax: 0086-21-56693614, E-mail: fenghougao@163.com;

Wei Liu, Ph.D., Shanghai General Hospital, Shanghai Jiao Tong University School of Medicine, Shanghai 200080, China. Phone: 0086-21-36126284; Fax: 0086-21-63243749, E-mail: bsjys@shsmu.edu.cn.

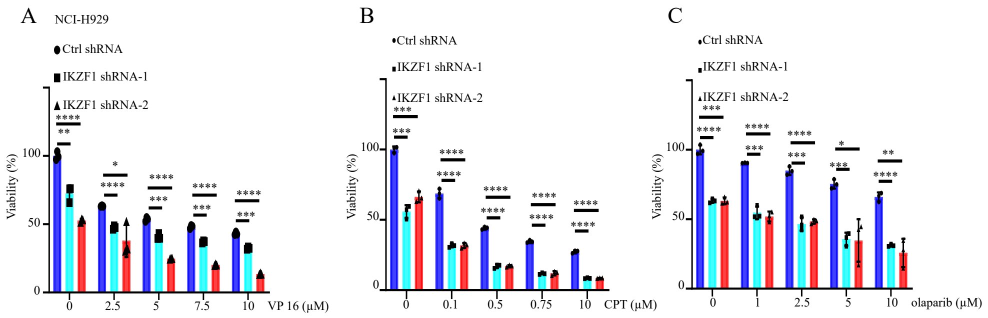

Liu et al. Figure S1

Fig. S1.

(A, B and C) NCI-H929 cells transfected with control shRNA or IKZF1 shRNA were treated with indicated doses of VP16, CPT and olaparib for 48 h, then assayed for the viability with CCK-8 kit. Viability relative to control shRNA is shown.

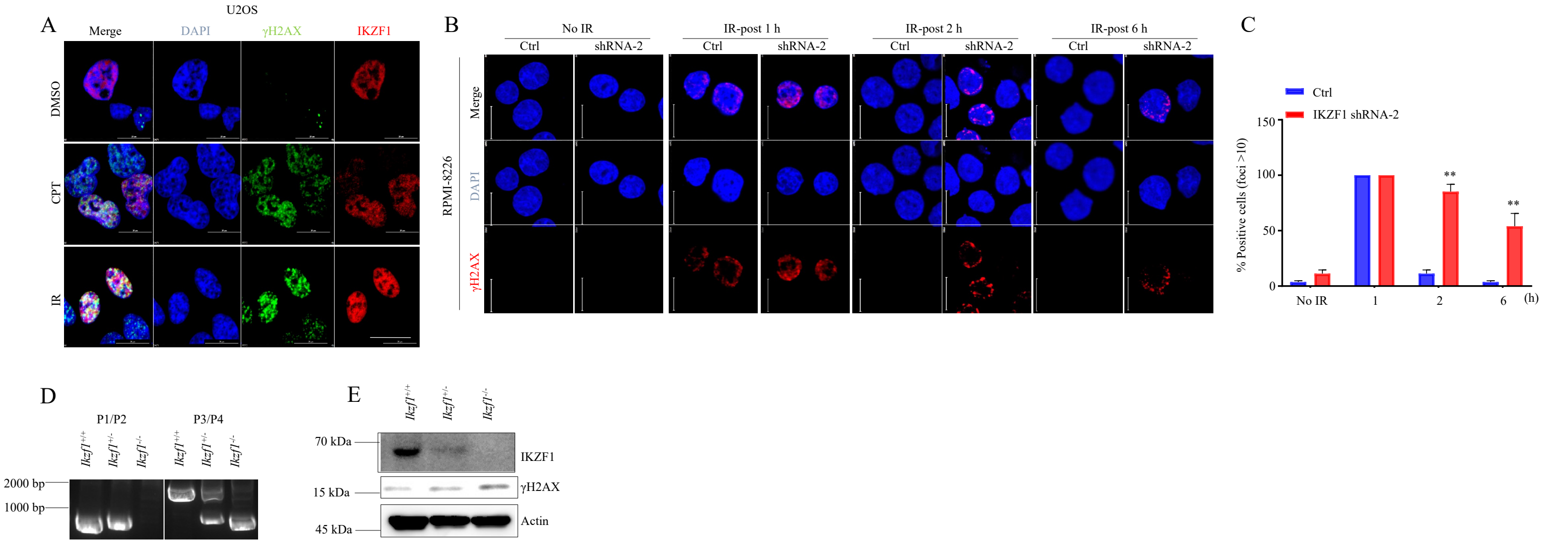

Liu et al. Figure S2

Fig. S2.

(A) Confocal microscopic analysis of IKZF1 foci formation upon DSBs. U2OS cells were tereated with CPT (100 nM) for 1 h or IR (5 Gy) and then fixed and immunostained with antibodies against IKZF1 and γH2AX. Scale bars: 20 μm. **(B, C)** RPMI-8226 cells stably expressing control shRNA and IKZF1 shRNA were subjected to IR (5Gy) and examined the γH2AX foci at the indicated time points **(B)**. Scale bar: 20 μm. Quantitation of γH2AX positive cells (foci >10) is shown **(C)**. **(D-F)** RPMI-8226 cells transfected with control shRNA and IKZF1 shRNA were treated with CPT (100 nM) or VP16 (1 μM) for 1 h, and then fixed and immunostained with antibodies against γH2AX foci **(D)**. Scale bars: 20 μm. Quantitation of γH2AX positive cells (foci >10) is shown **(F).** (G and H) Genotyping of IKZF1-knockout ($Ikzf1^{+/+}$, $Ikzf1^{+/-}$ and $Ikzf1^{-/-}$) mice by PCR (G). MEF cells from $Ikzf1^{+/+}$, $Ikzf1^{+/-}$ and $Ikzf1^{-/-}$ mice were treated with IR (5 Gy). The cellular extracts were collected 1 h after treatment for western blotting with indicated antibodies (H).

A

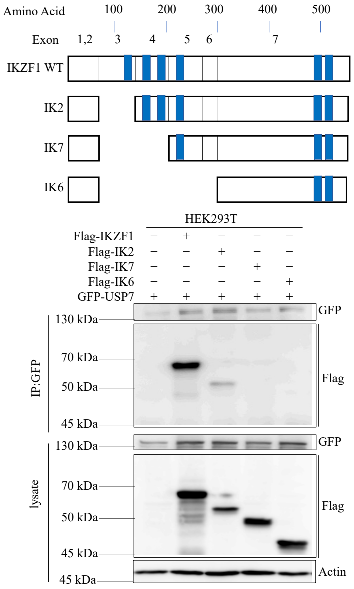

Liu et al. Figure S3

Fig. S3.

HEK293T cells transiently expressing GFP-USP7 were co-transfected with Flag-IKZF1 WT, Flag-IK2, Flag-IK7, or Flag-IK6, respectively. Cellular extracts were immunoprecipitated with anti-GFP antibody followed by IB with indicated antibodies. Zinc fingers are depicted in blue boxes.

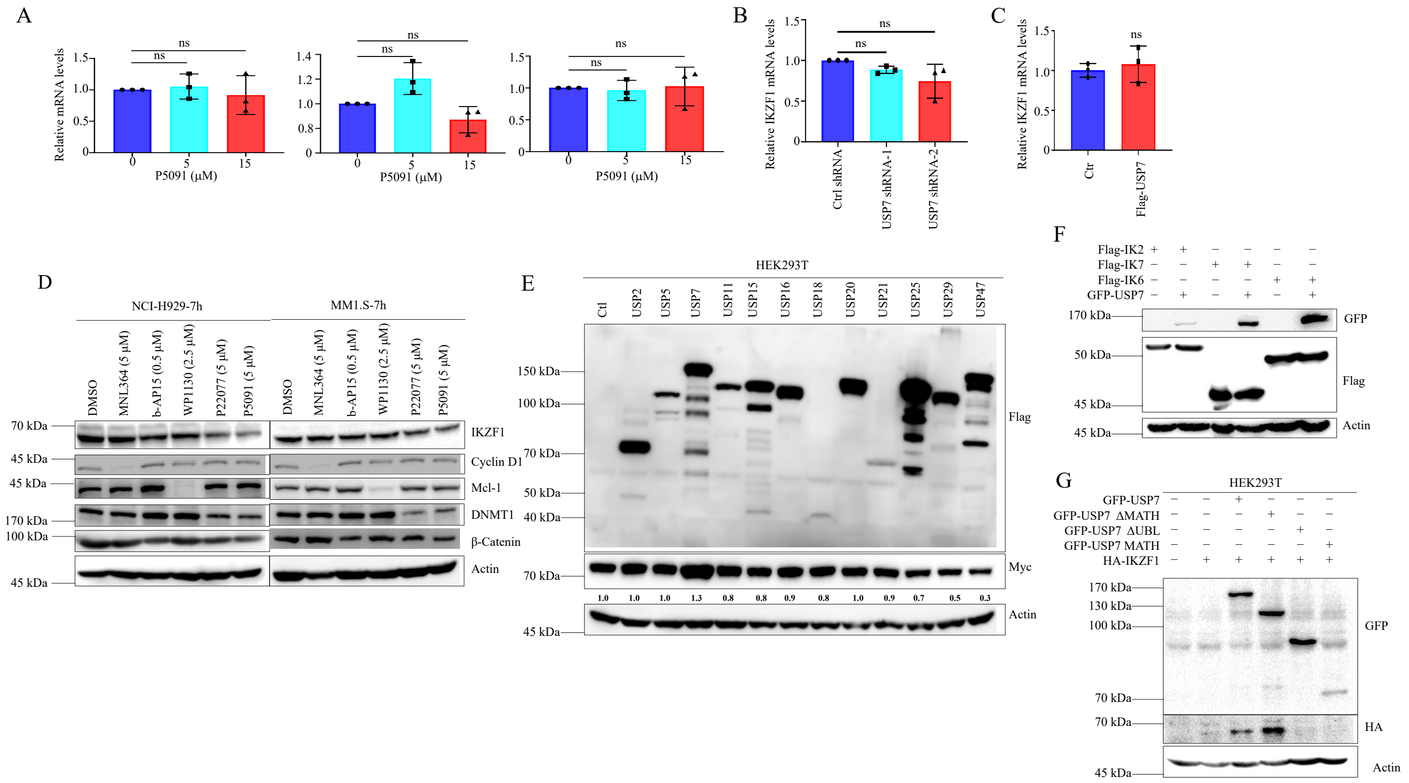

Liu et al. Figure S4

Fig. S4.

(A) RPMI-8226, MM1.S, and NCI-H929 cells were cultured in the absence or presence of the indicated doses of P5091 for 7 hours. IKZF1 mRNA were analyzed by qRT-PCR. **(B)** RPMI-8226 cells were transfected with the control shRNA or USP7 shRNAs, **(C)** or overexpressed with Flag-USP7. IKZF1 mRNA were analyzed by qRT-PCR. **(D)** NCI-H929 and MM1.S cells were treated with different DUB inhibitors for 7 h, cellular extracts were collected for western blotting with indicated antibodies. **(E)** HEK293T cells stably transfected with Myc-IKZF1 were transfected with Flag-tagged USPs. Cellular extracts were collected for western blotting with indicated antibodies. USP7 is indicated by the red dotted box. **(F)** HEK293T cells stably transfected with Flag-IKZF1 WT, Flag-IK2, Flag-IK7, or Flag-IK6 were co-transfected with or without GFP-USP7. Cellular extracts were collected for western blotting with the indicated antibodies. **(G)** HEK293T cells stably expressing HA-IKZF1 were cotransfected with GFP-USP7 WT, GFP-USP7 ΔMATH, or GFP-USP7 ΔUBL, or GFP-USP7 CD. Cellular extracts were collected for western blotting with indicated antibodies.

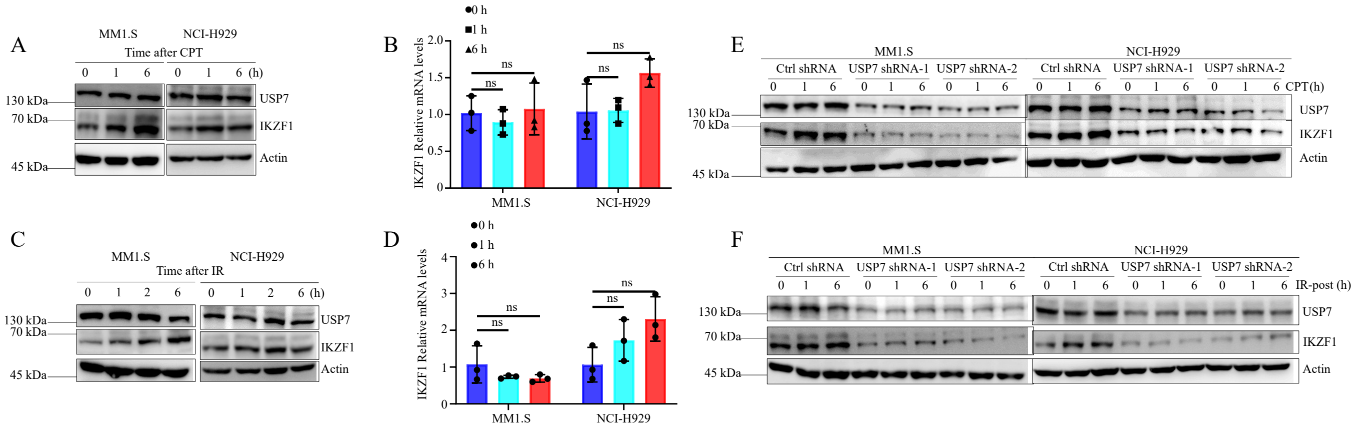

Liu et al. S Figure S5

Fig. S5.

(A-D) MM1.S and NCI-H929 cells exposed to CPT (100 nM) or IR (5 Gy) were harvested at the indicated time points. Cellular extracts were collected for western blotting with the indicated antibodies **(A and C)** and IKZF1 mRNA were analyzed by qRT-PCR **(B and D)**. Data represent the mean ± S.D. of biological triplicate experiments. *p*-values were calculated by Student's *t* test. **(E)** MM1.S or NCI-H929 cells stably expressing control shRNA and USP7 shRNA were subjected to CPT (100 nM) for 1 h, **(F)** or treated with IR (5 Gy) for indicated times. Cell lysates were subjected to western blotting with anti-USP7 and anti-IKZF1 antibodies.

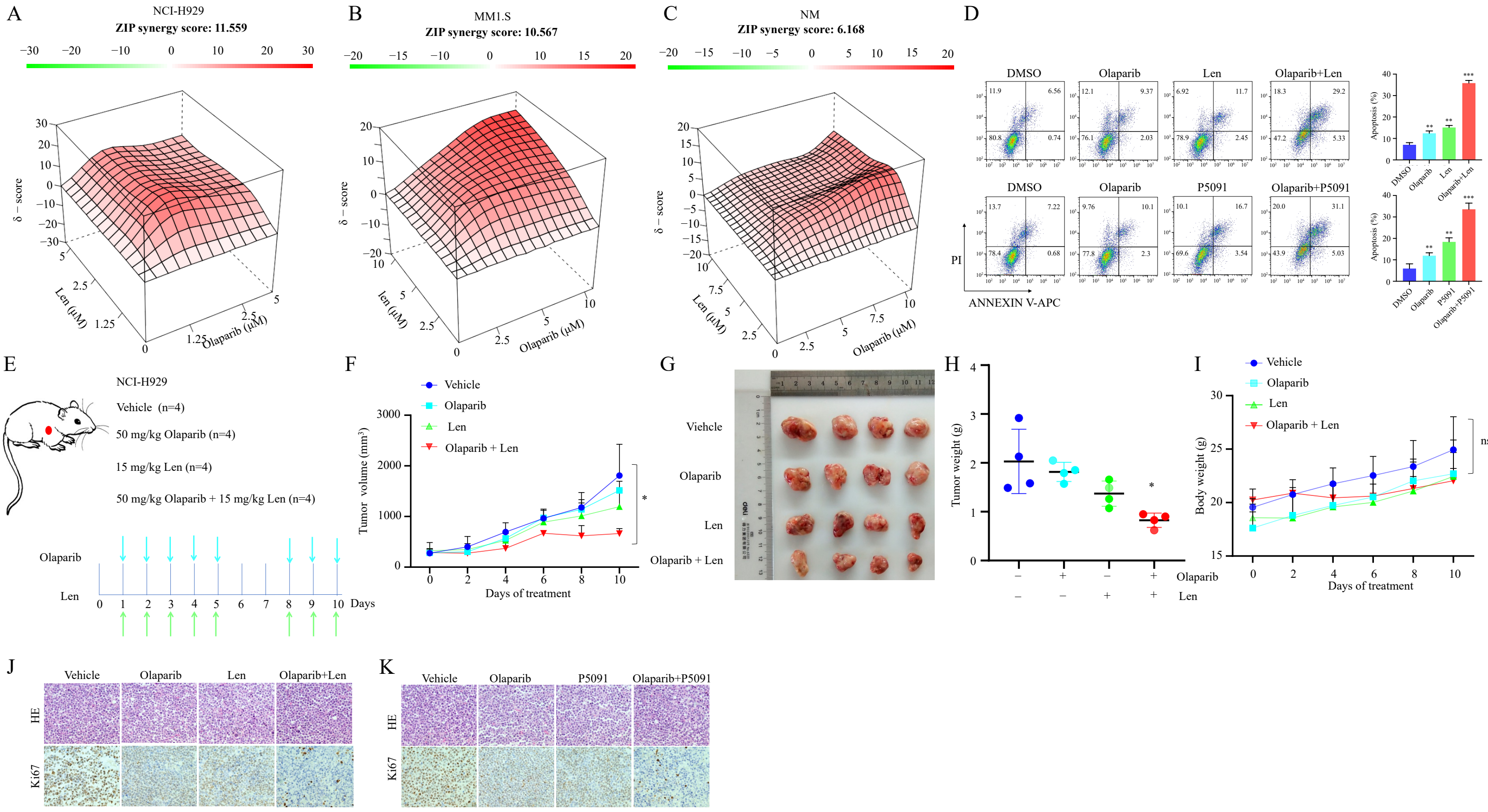

Liu et al. S Figure S6

Fig. S6.

Lenalidomide sensitizes multiple myeloma cells to PARPi in vitro and in vivo. (A-C) NCI-H929 (A), MM1.S (B), and normal BM mononuclear cells (C) were cultured in the control medium or in the presence of lenalidomide and/or olaparib for 48 h. The cell viability was determined by the CCK-8 kit. Data were analyzed online (https://synergyfinder.fimm.fi). (D) And the apoptosis was detected by the Annexin V-APC/PI apoptosis kit. (E-J) NCI-H929 cells were subcutaneously injected into the flank of NOD-SCID mice. Mice were treated with the vehicle, lenalidomide (15 mg/kg i.g.) and/or olaparib (50 mg/kg i.p.) (E). Mice tumor volume (F), tumor images (G), tumor weight (H), and body weight (I), and immunohistochemical (IHC) (J) were then assessed. (K) IHC of mice tumor (RPMI-8226 cells). Data are mean ± s.d. p-values were analyzed by two-way analysis of variance (ANOVA). $^*p < 0.05$, ns: no significant.
